# *HOX *genes in ovarian cancer

**DOI:** 10.1186/1757-2215-4-16

**Published:** 2011-09-09

**Authors:** Zoë L Kelly, Agnieszka Michael, Simon Butler-Manuel, Hardev S Pandha, Richard GL Morgan

**Affiliations:** 1 Postgraduate Medical School, Faculty of Health and Medical Sciences, University of Surrey, GU2 7WG, UK; 2 Royal Surrey County Hospital, Egerton Road, Guildford, Surrey, GU2 7XX, UK

**Keywords:** Ovarian, Cancer, HOX, Therapy, HXR9

## Abstract

The *HOX *genes are a family of homeodomain-containing transcription factors that determine cellular identity during development. Here we review a number of recent studies showing that *HOX *genes are strongly expressed in ovarian cancer, and that in some cases the expression of specific *HOX *genes is sufficient to confer a particular identity and phenotype upon cancer cells. We also review the recent advances in elucidating the different functions of *HOX *genes in ovarian cancer. A literature search was performed using the search terms *HOX *genes (including specific *HOX *genes), ovarian cancer and oncogenesis. Articles were accessed through searches performed in ISI Web of Knowledge, PubMed and ScienceDirect. Taken together, these studies have shown that *HOX *genes play a role in the oncogenesis of ovarian cancer and function in the inhibition of apoptosis, DNA repair and enhanced cell motility. The function of *HOX *genes in ovarian cancer oncogenesis supports their potential role as prognostic and diagnostic markers, and as therapeutic targets in this disease.

## Introduction

The *HOX *genes constitute a family of transcription factors that play key roles in embryonic development, especially in the patterning of the anterior to posterior axis, from the level of the hindbrain to the end of the spine. They are characterized by a highly conserved homeodomain region consisting of a 61-amino acid motif which enables HOX proteins to bind to specific regions of DNA in order to transcriptionally activate or repress target genes. HOX proteins can function as monomers or homodimers, although their target DNA binding affinities and specificities are enhanced by functioning as heterodimers or heterotrimers with members of the three-amino acid loop extension (TALE) family of co-factors, PBX and MEIS. *HOX *genes play fundamental roles during embryonic development, controlling anterior-posterior pattern formation, proliferation and differentiation [1]. In adult tissue, *HOX *genes have been implicated in a variety of biological pathways including homeostasis, cell differentiation and the maintenance of organ functioning [2].

In mammals, 39 *HOX *genes have been identified and organised into 4 paralogous clusters (A, B, C and D) located on 4 different chromosomes [3]. Each cluster contains 9-11 genes aligning in 13 paralogue groups based on homeobox sequence similarity and the position within a cluster. In embryonic development each *HOX *gene is expressed in a spatiotemporal pattern whereby the position of the *HOX *gene within a *HOX *cluster corresponds to its order of expression along the anterior to posterior axis. Thus for example, the 5' genes (paralogues 9-13) are involved in the differentiation of genitourinary structures in the lumbosacral region. *HOX *expression in adult tissues often reflects embryonic expression, and different cancers show distinct changes from normal adult expression [4]. These include temporospatial deregulation where *HOX *gene expression in a tumour of a specific tissue is temporospatially different from the expression seen in the normal tissue, and/or the increased expression of *HOX *genes that are usually expressed at lower levels in the normal tissue. In addition, epigenetic changes can result in the down-regulation or silencing of certain *HOX *genes which normally function as tumour suppressors. Numerous studies have demonstrated deregulated *HOX *gene expression in cancer including lung, prostate, breast, colon, bladder and thyroid cancer [5-9] and also in ovarian cancer.

### *HOX *genes and Ovarian Cancer

**Figure 1:**
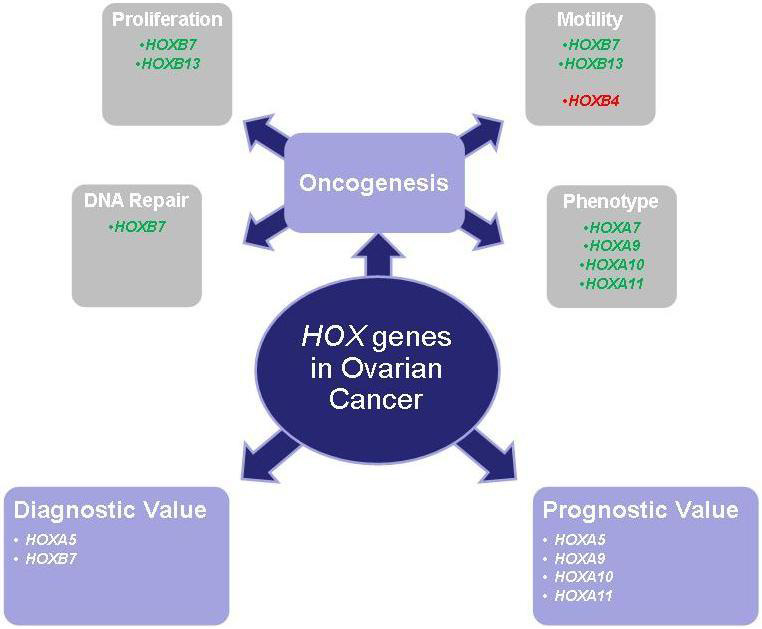
**Role of *HOX *genes in ovarian cancer**. A summary of the possible involvement of *HOX *genes in the cell biology of ovarian cancer and their potential use as clinical markers for the disease. Up regulated genes are shown in green, down regulated genes are shown in red.

During development of the female reproductive system four *HOX *genes, *HOXA9, HOXA10, HOXA11*, and *HOXA13 *are expressed uniformly along the Müllerian duct axis, although in adults their expression becomes spatially restricted to particular organs. *HOXA9 *becomes expressed in the fallopian tubes, *HOXA10 *is expressed in the developing uterus, *HOXA11 *in the lower uterine segment and cervix and *HOXA13 *in the upper vagina [10]. Their expression is thought to be important in preserving a high level of developmental plasticity of the female reproductive system due to the dramatic structural and functional changes which occur during the oestrous cycle and pregnancy [10]. It is thought that inappropriate expression of these genes is an early step in epithelial ovarian neoplasia as they induce aberrant epithelial differentiation. A study by Cheng *et al. *(2005) [11] used immunohistochemical analysis of biopsied tissue to show that the *HOX *genes which normally regulate Müllerian duct differentiation were not expressed in normal ovarian surface epithelia (OSE) but were expressed in epithelial ovarian cancers (EOCs). The *HOX *expression pattern appears to determine the histological identity of EOCs with serous papillary, endometriod and mucinous tumours showing overexpression of *HOXA9*, *HOXA10 *and *HOXA11*, respectively [11]. Another *HOX *gene, *HOXA7*, was reported to be aberrantly expressed both at the RNA level (by quantitative PCR) and at the protein level (by immunohistochemistry using an anti-HOXA7 antibody) in ovarian cancer tissues which display Müllerian-like characteristics, however little or no expression was found in undifferentiated ovarian carcinomas and normal OSE [12]. When expressed ectopically in an undifferentiated ovarian mouse tumour, HOXA7 was shown to promote the ability of *HOXA9*, *HOXA10 *and *HOXA11 *to induce Müllerian differentiation rather than induce lineage specificity itself [11,12]. These results suggest *HOXA7 *plays a role in epithelial differentiation of OSE in ovarian epithelial cancers, although Ota *et al. *(2007) found *HOXA7 *overexpression in all ovarian carcinomas tested, including undifferentiated subtypes. This could suggest that *HOXA7 *is associated with Müllerian differentiation but is not sufficient to maintain it (Figure 1).

Several other studies have reported altered *HOX *gene expression in ovarian carcinomas when comparing to normal OSE [12-14] however, there are some discrepancies. Naora *et al. *(2001) [15] found *HOXB7 *was expressed at higher levels in ovarian carcinomas compared to normal OSE. This was supported by a later study by Yamashita *et al. *(2006) [13] who created an expression profile of HOX genes in ovarian-derived samples. In this study overexpression of 16 *HOX *genes in ovarian cancer cell lines were found, the most common being *HOXB7*, *HOXA13 *and *HOXB13*. Overexpression varied between cell lines but of these 16 genes, *HOXA10*, *A13*, *B4*, *B7*, *B13 *and *C13 *showed little or no expression in normal samples (Table 1). It should be noted though that both of these studies examined the expression of *HOX *genes at the RNA level only by quantitative PCR.

**Table 1:** Over expression of *HOX *genes in ovarian cancer cell lines using RT-PCR

	Cell Line				
***HOX *gene**	**SKOV-3**	**CaOV3**	**JHOC-6**	**SMOV2**	**ES-2**

A3	-	-	-	2.0	-
A4	-	-	-	-	2.0
A7	-	2.1	-	-	-
A10	12.1	13.3	3.5	-	12.6
A13	> 300	> 300	> 300	> 300	> 300

B2	9.2	-	-	-	4.1
B3	6.6	-	-	-	-
B4	267	44.9	-	141.8	91.8
B5	14.9	-	-	-	-
B6	9.4	-	-	-	-
B7	> 300	> 300	> 300	> 300	> 300
B8	47.6	-	3.2	-	-
B9	6.1	3.1	3.0		5.6
B13	> 300	> 300	> 300	> 300	> 300

C13	-	3.3	-	-	-

D13	3.6	-	-	-	-

Shows over expression of *HOX *genes in ovarian cancer cell lines by RT-PCR. Numbers indicate folds of expression level of each *HOX *gene in cancer cells compared with that in normal samples. After ref [13].

Slightly different results were found in a more recent study by Hong *et al. *(2010) [16]. In this study the expression of 36 *HOX *genes in ovarian cancer cell lines and tissues were compared to normal ovarian tissue, revealing a difference in expression of 11 *HOX *genes (*HOXA7*, *B3*, *B4*, *B6*, *C10*, *C11*, *D1*, *D3*, *D10*, *D11 *and *D13*). Of these 11 genes, *HOXB4 *was the only *HOX *gene showing significantly higher levels of expression in ovarian cancer cell lines than in normal ovarian tissue (p = 0.029). Its expression was confirmed at protein level by Western blot analysis, with exclusive expression in all 4 ovarian cancer cell lines and all 7 ovarian cancer tissue samples and not in the normal ovarian tissues. *HOXB4 *has also been implicated as a cancer-related gene in other malignancies including breast cancer, leukaemia, osteosarcoma and lung cancer [17-20].

### Function of *HOX *genes in ovarian cancer

#### Tumour Growth

In addition to phenotypic determination, *HOX *genes are thought to play a role in the oncogenesis of ovarian cancer. *HOXB7 *overexpression in immortalised OSE cells was shown to upregulate basic fibroblast growth factor (bFGF). bFGF is a potent mitogenic and angiogenic factor, although in this study it should be noted that the majority (95%) of bFGF protein was intracellular and a relatively limited amount may therefore be available for cell to cell signalling [15]. *HOXB13 *has also been shown to enhance the proliferation of ovarian cancer cell lines SKOV-3 and OVCAR5 *in vivo*, and to promote the growth of a mouse ovarian cancer cell line *in vivo *and *in vitro *[21]. This oncogenic function of *HOXB13 *is thought to require activated *ras*, as *HOXB13 *promoted tumourgenesis in ovarian cancer cell lines containing genetic alterations in *p53*, *myc *and *K-ras *but not in cell lines containing genetic alterations in *p53*, *myc *and *Akt*. In this ras activated cell line, *HOXB13 *also conferred resistance to tamoxifen-mediated apoptosis suggesting a pro-survival role in ovarian cancer.

*HOXA10 *is also strongly expressed at the protein level in ovarian clear cell adenoarcinomas (OCCA, 20/29 cases) [11,22] but not expressed in normal ovarian epithelia, ovarian serous adenocarcinomas, or endometrial cysts [22]. When over expressed in the human OCCA derived cell line ES-2, *HOXA10 *resulted in increased proliferation, and a two-fold increase in cell invasion as measured by a *Matrigel *invasion assay. Correspondingly, its expression in tumour samples correlates with poor survival. The 5-year survival rate in patients with *HOXA10 *positive tumours was only 30%, but it was 55.6% in the *HOXA10 *negative group [22].

#### Cell motility and spreading

In addition to promoting proliferation, *HOXB7 and HOXB13 *are also thought to be associated with the invasive characteristics of ovarian cancer cells. This invasive ability was investigated in a study by Yamashita *et al. *(2006) [13] using the invasive cancer cell line SKOV-3, which overexpresses *HOXB7 *and *HOXB13*. Antisense *HOXB7 *and *HOXB13 *fragments were introduced into SKOV-3 cells which results in an 85% and 50% reduction of motility, respectively, suggesting a role in cancer cell invasion. However, as invasion was not completely suppressed the function of these *HOX *genes may be redundant and complemented by closely related genes such as *HOXA13*, which was also overexpressed in this cell line.

Invasive EOC cell lines also show overexpression of *HOXA4 *compared to non-invasive cell lines, suggesting a possible role for *HOXA4 *in promoting migration and invasion. However a number of studies have indicated that the role of *HOXA4 *could be invasion-suppressive as siRNA-mediated knockdown of *HOXA4 *enhanced cellular motility in normal OSE cells treated with EGF [23], although it had no affect on the basal levels of migration in the absence of EGF. This finding was supported in part by Klausen *et al. *(2009) [24] where knockdown of *HOXA4 *in OVCAR-3 and OVCAR-8 cells increased migration (although not *Matrigel *invasion). *HOXA4 *knockdown also reduced cell-cell adhesion and β1 integrin protein level within cell colonies, suggesting β1 integrin has a role in mediating these changes. Intriguingly, these changes in protein level are not reflected at the RNA level, indicating that the effect of *HOXA4 *on β1 integrin and hence cell motility may be through an indirect mechanism. Taken together these findings suggest that *HOXA4 *is indeed primarily a suppressor of invasion, and it is possible then that the increased *HOXA4 *expression observed in invasive cell lines may be linked to a tumour-suppressive response.

#### DNA Repair

*HOXB7 *is one of the *HOX *genes which shows a markedly higher expression in ovarian cancer cell lines compared to normal ovarian epithelia [15] and promotes growth in ovarian epithelial cells [11], but it also plays a novel role in DNA double-strand break repair through interacting with proteins that act as genomic caretakers, including members of the DNA-dependent protein kinase haloenzyme, Ku70, Ku80 and DNA-PK_cs _[25]. Binding of HOXB7 to such haloenzymes endogenously and exogenously increased DNA repair through poly(ADP) ribose polymerase (PARP) activity. Different *HOXB7 *expressing breast cancer cell lines exposed to ionising radiation (IR) showed enhanced end-joining product formation and enhanced double-strand break repair and non-malignant cell lines that were transfected with a HOXB7 expression vector developed increased resistance to killing by IR. Correspondingly, chromosomal damage was reduced following IR and less residual damage was seen in cells expressing *HOXB7*, an effect which could be reversed by *HOXB7 *silencing. These findings suggest that HOXB7 could be a potential target for therapies that enhance IR cell killing.

### Targeting HOX genes

As the function of the *HOX *genes is partly based on the binding of HOX proteins to the TALE homeobox set of co-factors, PBX and MEIS, the oncogenic features of the aberrantly expressed HOX proteins could be impaired by targeting these co-factors. PBX and MEIS have been found extensively expressed both nuclear and cytoplasmically in ovarian carcinomas but only MEIS 1 and 2 are expressed in the nucleus of normal epithelia [26]. This suggests that these co-factors are important in ovarian carcinogenesis most probably by potentiating the function of aberrantly expressed HOX proteins.

A peptide called HXR9 has been designed to target the interaction between HOX and PBX. This drug has been shown to retard tumour growth and induce apoptosis in the ovarian cancer cell line SKOV-3 [27], a cell line which shows highly deregulated expression of certain *HOX *genes. However, this effect was not seen in the OV-90 cell line which does not show *HOX *gene deregulation. HXR9 treatment has proven to be successful in other malignancies showing *HOX *gene deregulation including melanoma [28], myeloma [29], renal cancer [30] and lung cancer [31]. These results show that PBX/HOX binding is a potential target in ovarian cancer.

### *HOX *genes as potential biomarkers and prognostic markers

Circulating autologous antibodies to tumour antigens are potential diagnostic biomarkers for the detection of early stage cancers. A systematic search for an ovarian tumour antigen was undertaken by Naora *et al *(2001) [15]. In this study, the SEREX methodology (serological identification of antigens by recombinant expression cloning) was used to screen tumour cDNA expression libraries with ovarian cancer patient serum. *HOXB7 *was one of the *HOX *genes found in this screen, with significant serological reactivity to HOXB7 in 13 of the 39 ovarian cancer patients and in 1 healthy female, suggesting the detection of anti-HOXB7 antibodies could act as a possible diagnostic tool [13,15]. However, further research using larger sample number sizes and correlation studies of titre of anti-HOXB7 with disease stage is needed. Similar methodology applied to the serum of patients with serous ovarian carcinomas identified the *HOXA7 *gene as a potential biomarker of this disease [12], and *HOXA10 *as a promising prognostic marker for OCCA as its over expression is strongly correlated with poor survival and not expressed in normal OSE [22].

*HOX *genes showing markedly higher expression in ovarian cancer cell lines and cancer tissue specimens compared to the normal ovaries also have the potential of acting as prognostic markers, these include *HOXB4 *[16] and *HOXB7 *[25]. In microarray analysis of ovarian tissues, *HOXA5 *and *HOXA9 *were both shown to be overexpressed [32]. *HOXA5 *has been found to act as a tumour suppressor in breast tissue by transactivating the *p53 *gene [7] to induce apoptosis by p53-dependent and p53-independent mechanisms [33]. A possible tumour-suppressor role for *HOXA5 *is also supported by low *HOXA5 *expression in breast [7] and lung cancer [34] tissues which is thought to be mediated by methylation of the CpG island located on the 5' end of the *HOXA5 *gene [7].

In addition to expression profiling, epigenetic alterations associated with ovarian carcinogenesis have been studied [35-37]. The most common molecular alteration in human neoplasia is DNA methylation [38] and this could possibly act as a prognostic marker. Fiegl *et al. *(2008) [39] analysed the DNA methylation of 71 genes in 22 ovarian cancers and 18 non-neoplastic samples and identified the best discriminators between cancer and non-neoplastic tissue as being *HOXA10 *and *HOXA11*. In particular *HOXA11 *methylation was strongly associated with poor outcome, suggesting a possible role for DNA methylation as a prognostic marker in ovarian cancer. A more recent study has shown that increased expression of *HOXA10 *is present in ovarian carcinomas as a result of promoter hypomethylation of *HOXA10 *[40]. This could also act as a prognostic factor in ovarian cancer as well as a possible therapeutic target, for example by using drugs that can reverse epigenetic changes.

## Conclusions

*HOX *gene function is associated with the oncogenesis of ovarian cancer, having a proven role in proliferation, cell migration and DNA repair, although the exact mechanisms and pathways involved require further study. Their oncogenic function together with the observed differences in expression between normal ovarian tissue and ovarian cancers suggest a potential diagnostic and prognostic value for some *HOX *genes. This potential is broad ranging as the oncogenic and/or tumour suppressor functions of *HOX *genes indicate numerous possible influences on the subtype of ovarian cancer, its aggressiveness and the likelihood of metastasis and angiogenesis, together with its response to at least certain types of therapy, including radiotherapy. The expression of individual *HOX *genes or groups of *HOX *genes in tumours, circulating cells or cells in the ascites, or the presence of HOX proteins in a range of biological fluids are all potential sources of valuable clinical information.

It is also apparent that *HOX *genes are potential therapeutic targets, as are the mechanisms which regulate HOX protein function, such as the HOX/PBX heterodimer which upon disruption has shown anti-tumour effects in a variety of cancers [27,28,30,31]. These treatments could be used alone or in combination with other treatment modalities to increase tumour susceptibility; for example treating ovarian tumours showing highly elevated levels of HOXB7 with HXR9 could sensitise cells to killing by ionising radiation. This approach may also be valuable for treatment of drug-resistant cancers, especially if *HOX *expression in the tumour indicates a high level of *HOX *deregulation.

## Competing interests

The authors declare that they have no competing interests.

## Authors' contributions

ZLK and RGLM wrote the review. AM, HSP and SBM provided advice on which literature to include and which studies were most relevant. All authors have read and approved the final manuscript.
